# Metabolic stone workup abnormalities are not as important as stone culture in patients with recurrent stones undergoing percutaneous nephrolithotomy

**DOI:** 10.1007/s00240-023-01422-w

**Published:** 2023-03-13

**Authors:** Asmaa E. Ahmed, Hassan Abol-Enein, Amira Awadalla, Ahmed A. Shokeir, Omar A. El-Shehaby, Ahmed M. Harraz

**Affiliations:** 1 Botany Department, Faculty of Science, Mansoura University, Mansoura, Egypt; 2 Urology Department, Urology and Nephrology Center, Mansoura University, Mansoura, 35516 Egypt; 3 Center of Excellence for Genome and Cancer Research, Urology and Nephrology Center, Mansoura University, Mansoura, Egypt

**Keywords:** Stone recurrence, Metabolic workup, Urinary tract infection, Stone culture, 24 h urine test, Nephrolithiasis

## Abstract

To investigate the association between metabolic urinary abnormalities and urinary tract infection (UTI) and the stone recurrence status in patients undergoing percutaneous nephrolithotomy (PCNL). A prospective evaluation was performed for patients who underwent PCNL between November 2019 and November 2021 and met the inclusion criteria. Patients with previous stone interventions were classified as recurrent stone formers. Before PCNL, a 24 h metabolic stone workup and midstream urine culture (MSU-C) were done. Renal pelvis (RP-C) and stones (S-C) cultures were collected during the procedure. The association between the metabolic workup and UTI results with stone recurrence was evaluated using univariate and multivariate analyses. The study included 210 patients. UTI factors that showed significant association with stone recurrence included positive S-C [51 (60.7%) vs 23 (18.2%), p < 0.001], positive MSU-C [37 (44.1%) vs 30 (23.8%), p = 0.002], and positive RP-C [17 (20.2%) vs 12 (9.5%), p = 0.03]. Other factors were mean ± SD GFR (ml/min) (65 ± 13.1 vs 59.5 ± 13.1, p = 0.003), calcium-containing stones [47 (55.9%) vs 48 (38.1%), p = 0.01], median (IQR) urinary citrate levels (mg/day) [333 (123–512.5) vs 221.5 (120.3–412), p = 0.04], and mean ± SD urinary pH (6.1 ± 1 vs 5.6 ± 0.7, p < 0.001). On multivariate analysis, only positive S-C was the significant predictor of stone recurrence (odds ratio: 9.9, 95% confidence interval [CI] (3.8–28.6), p < 0.001). Positive S-C, and not metabolic abnormalities, was the only independent factor associated with stone recurrence. A focus on preventing UTI might prevent further stone recurrence.

## Introduction

Urolithiasis represents a prevalent pathology that urologists encounter in everyday practice. Its significance stems from the high volume of cases, costly and painful renal colic episodes, and the requirements for updated healthcare facilities. One important aspect of urinary stones is the high rate of recurrence in both the pediatric and adult populations [1, 2]. The recurrence rates were 11, 20, 31, and 39% at 2, 5, 10, and 15 years, respectively [3]. Various definitions have been proposed for stone recurrence that can be classified as either symptomatic or radiological recurrence [4]. Stone recurrence is a worthy investigation as it adds to the burden of repeated emergency and outpatient visits, frequent imaging, various interventions, and the need for continuous follow-up [5].

Risk factors and prevention of stone recurrence have been the focus of a plethora of published articles. Identified potential risk factors included younger age, male sex, higher body mass index (BMI), positive family history, pregnancy, a history of uric acid, struvite or brushite stones, non-calcium oxalate monohydrate stones, urine pH, and the presence of diabetes mellitus [6, 7]. A nomogram has been previously developed to predict the recurrence risk based on a group of clinical factors [3].

While 24 h urinary metabolic evaluation has been recommended to guide the therapy to prevent stone recurrence [8, 9], recent reports questioned its role [5, 10–12]. In addition, another unforeseen parameter that might potentially affect stone recurrence is the presence of urinary tract infection (UTI) particularly in staghorn stones [13, 14]. Bacterial infection has been shown to promote the growth and aggregation of calcium oxalate crystals [15]. In this context, this study was designed to evaluate the association between the recurrent stone status and the presence of active UTI represented by positive midstream urine culture (MSU-C), renal pelvis culture (RP-C), or stone culture (S-C).

## Materials and methods

### Study design

A prospective evaluation of patients who underwent percutaneous nephrolithotomy (PCNL) in a tertiary referral center was performed between November 2019 and November 2021. Informed consent was taken before enrollment in the study. The study protocol has been reviewed and approved by the local ethical committee and the institutional review board. Patients with stents or indwelling catheters or those who fail to provide a 24 h urine collection were excluded from the study. Other exclusion criteria included the presence of medical conditions that contribute to stone formation (hyperparathyroidism or renal tubular acidosis), or anatomical abnormalities (UPJ stenosis or horseshoe kidney).

### Measurements and intervention

Upon admission, patients' demographic data were recorded, including gender, associated comorbidities, and BMI. Serum tests included creatinine, sodium, potassium, magnesium, calcium, phosphorus, and albumin. Patients were asked to provide 24 h urinary collection to undergo a full metabolic workup that included 24 h urinary calcium, phosphorus, oxalate, citrate, and uric acid. In addition, urine pH was measured, and the glomerular filtration rate (GFR) was calculated using the 24 h urine volume, serum, and urinary creatinine. Urinary constituents were analyzed based on raw numbers and by laboratory standards. The cut-off values of hypercalciuria, hyperuricosuria, hyperoxaluria and hypocitraturia were 200 mg/day, 750 mg/day, 45 mg/day, and 320 mg/day, respectively.

According to our protocol, any patient with positive pre-operative MSU-C receives the appropriate antibiotic 3–7 days before PCNL to prevent postoperative infectious complications. A routine third-generation cephalosporin was administered one hour before the surgery if MSU-C was negative. All patients underwent PCNL in the prone position after a ureteral catheter fixation. The caliceal puncture was done under fluoroscopic guidance and mechanical dilatation was done using Alkene’s metal dilators. Stone disintegration was accomplished using mechanical or laser disintegration. Postoperative nephrostomy tube placement and ureteral versus JJ stent placement were left to the discretion of the surgeon. Before stone disintegration, a renal pelvis urine sample was obtained and sent separately for culture (RP-C). In addition, fragments of stones were sent for stone culture (S-C) according to Tavichakorntrakool et al. method [16] and biochemical analysis with infrared spectrophotometry (Fourier-transform infrared spectroscopy (FTIR) 2000, Perkin-Elmer Co., U.S.A*)*.

### Outcome

The primary outcome of the study was to identify the relationship between ipsilateral renal stone intervention history and both the metabolic workup, and results of MSU-C, RP-C, and S-C. Any patient with a previous history of PCNL or retrograde intrarenal surgery either in our hospital or elsewhere with documented stone-free status or the presence of clinically insignificant residual fragments (< 3 mm) at the time of hospital discharge and completed at least 6 months free period was considered a recurrent stone former. In addition, patients with no history of any stone intervention were considered primary stone formers. Cohen’s Kappa was used to describe the level of agreement between each pair of RP-C, S-C, and MSU-C. The level of agreement is considered excellent, fair to good, and poor for Kappa levels more than 0.75, 0.4–0.75, and less than 0.4, respectively.

### Statistical analysis

Numeric data were displayed as mean ± SD or median (IQR) according to parametric distribution and the significance level was calculated using the Student t or Mann–Whitney U tests, respectively. Categorical variables were presented as percentages in each category and were compared using the Chi-square test. Factors with a significance level of < 0.05 on univariate analysis were entered into a multivariate logistic regression model to identify the independent predictors of stone recurrence. To avoid multicollinearity in logistic regression model covariates, 3 distinct models were constructed using MSU-C, RP-C, and S-C separately. The area under the curve (AUC) was calculated for each model and compared to select the final model with the best performance. The statistical analysis was performed using R programming language version 4.1.2.

## Results

### Demographics

A total of 210 patients were included during the study period of which 84 (40%) patients had a history of stone intervention with a free intervening period. 99 (47.1%) patients had positive findings in either the MSU-C, RP-C, or S-C. Most of our patients were obese with a mean BMI of 32.3 ± 6.7 and 132 (62.9%) were females. The mean ± SD GFR was 61.7 ± 13.4 ml/min with none of our patients had chronic renal failure. The most common stone type detected was uric acid stones in 99 (47.1%) patients while Ca oxalate stones were found in 62 (29.5%) patients. *Staphylococcus aureus* (*S. aureus*) was the most common organism found in S-C and RP-C in 33 (15.7%) and 15 (7.1%) patients, respectively. On the other hand, *Escherichia coli* (*E. coli*) was the most common organism isolated from MSU-C in 39 (18.6%) patients. Table 1 demonstrated the characteristics of patients, stones, and the results of metabolic workup.

**Table 1:** Patients and stone characteristics and the results of the metabolic workup

Variable	Value
Age	49.6 ± 12.2
Gender	
Female	132 (62.9%)
Body mass index	32.3 ± 6.7
DM	
Yes	37 (17.6%)
GFR (ml/min)	61.7 ± 13.4
Hypertension	
Yes	62 (29.5%)
Stone density (Hounsfield units)	579 (443.5–1004)
Stone size (mm)	44 (20.9–96)
Stone type	
Ca oxalate	62 (29.5%)
Ca phosphate	12 (5.7%)
Uric acid	99 (47.1%)
Magnesium ammonium phosphate	6 (2.9%)
Ca oxalate and uric acid	13 (6.2%)
Cystine	10 (4.8%)
Ca Oxalate and Ca phosphate	8 (3.8%)
Midstream urine culture (MSU-C)	
*E. coli*	39 (18.6%)
*S. aureus*	13 (6.2%)
*E. faecalis*	4 (1.9%)
*P. aeruginosa*	3 (1.4%)
*K. pneumonia*	8 (3.8%)
Negative	143 (68.1%)
Renal pelvis culture (RP-C)	
*E. coli*	7 (3.3%)
*S. aureus*	15 (7.1%)
*E. faecalis*	4 (1.9%)
*P. aeruginosa*	3 (1.4%)
*K. pneumonia*	1 (0.5%)
*S. epidermidis*	1 (0.5%)
Negative	179 (85.2%)
Stone culture (S-C)	
*E. coli*	11 (5.2%)
*S. aureus*	33 (15.7%)
*E. faecalis*	10 (4.8%)
*P. aeruginosa*	10 (4.8%)
*K. pneumonia*	2 (1%)
*S. epidermidis*	8 (3.8%)
Negative	136 (64.8%)
Serum levels	
Albumin (g/dL)	4.5 ± 0.6
Calcium (mg/dL)	9.3 ± 0.9
Creatinine (mg/dL)	0.5 ± 0.2
Potassium (mmol/L)	4.3 ± 0.5
Magnesium (mg/dL)	2.3 ± 0.3
Sodium (mmol/L)	138.2 ± 4.1
Phosphate (mg/dL)	4.8 ± 0.9
24 h urine test (mg/day)	
Calcium	230.5 (210–400)
Citrate	288.6 (122–453.5)
Creatinine, mg/dL	66.4 ± 8.9
Oxalate	22 (15–45.8)
Phosphate	222 (201–321)
Uric acid	399 (222.5–607.5)
pH	5.8 ± 0.9
Hypercalciuria (> 200 mg/day)	
Yes	170 (81%)
Hyperoxaluria (> 45 mg/day)	
Yes	58 (27.6%)
Hyperuricosuria (> 750 mg/day)	
Yes	23 (11%)
Hypocitraturia (< 320 mg/day)	
Yes	119 (56.7%)
Recurrence	
Yes	84 (40%)

Data are described as mean ± SD or median (IQR) based on the parametric distribution

### Multivariate logistic regression models

Urinary tract infection factors that showed significant association with stone recurrence included positive S-C [51 (60.7%) vs 23 (18.3%), p < 0.001], positive MSU-C [37 (44.1%) vs 30 (23.8%), p = 0.002], positive RP-C [17 (20.2%) vs 12 (9.5%), p = 0.03]. Other factors were mean GFR ± SD (ml/min) (65 ± 13.1 vs 59.5 ± 13.1, p = 0.003), calcium-containing stones [47 (55.9%) vs 48 (38.1%), p = 0.01], median (IQR) urinary citrate levels (mg/day) [333 (123–512.5) vs 221.5 (120.1–412), p = 0.04], and mean ± SD urinary pH (6.1 ± 1 vs 5.6 ± 0.7, p < 0.001). Data are presented in Table 2.

**Table 2:** Univariate analysis for predictors of stone recurrence

	No recurrence	Recurrence	P-value
Age	49.7 ± 11.7	49.3 ± 13	0.8
Gender			0.5
Female	77 (61.1%)	55 (65.5%)	
DM			0.4
Yes	20 (15.9%)	17 (20.2%)	
GFR (ml/min)	59.5 ± 13.1	65 ± 13.1	0.003
Hypertension			0.1
Yes	42 (33.3%)	20 (23.8%)	
Body mass index	32.9 ± 6.7	31.3 ± 6.6	0.08
Obesity (BMI > 30)			0.09
Yes	89 (70.6%)	50 (59.5%)	
Stone size (mm)	41.8 (21.8- 91.5)	46.5 (20–105)	0.3
Stone type			0.01
Ca-containing	48 (38.1%)	47 (55.9%)	
MSU-C			0.002
Positive	30 (23.8%)	37 (44.1%)	
RP-C			0.03
Positive	12 (9.5%)	17 (20.2%)	
S-C			< 0.001
Positive	23 (18.2%)	51 (60.7%)	
Serum			
Albumin (g/dL)	4.5 ± 0.6	4.4 ± 0.6	0.2
Calcium (mg/dL)	9.2 ± 0.9	9.4 ± 0.8	0.09
Creatinine (mg/dL)	0.5 ± 0.1	0.5 ± 0.2	0.3
Potassium (mmol/L)	4.3 ± 0.5	4.4 ± 0.5	0.3
Magnesium (mg/dL)	2.4 ± 0.3	2.3 ± 0.3	0.1
Sodium (mmol/L)	138.3 ± 4.1	138.2 ± 4.1	0.9
Phosphate (mg/dL)	4.8 ± 0.8	4.9 ± 1	0.4
24 h urine test (mg/day)			
Calcium	222 (210–350.8)	231 (210–462.5)	0.5
Citrate	221.5 (120.3–412)	333 (123–512.5)	0.04
Creatinine, mg/dL	66.2 ± 8.4	66.6 ± 9.7	0.8
Phosphate	222 (210–321)	222 (200–321)	0.6
Uric acid	500.0 (224–613.3)	281.5 (222–566.3)	0.3
Oxalate	22 (14.0–44)	25.5 (16.5–50.3)	0.2
Urine pH	5.6 ± 0.7	6.1 ± 1	< 0.001
Hypercalciuria (> 200 mg/day)			0.7
Yes	103 (81.7%)	67 (79.8%)	
Hyperoxaluria (> 45 mg/day)			0.2
Yes	31 (24.6%)	27 (32.1%)	
Hyperuricosuria (> 750 mg/day)			0.7
Yes	13 (10.3%)	10 (11.9%)	
Hypocitraturia (< 320 mg/day)			0.03
Yes	79 (62.7%)	40 (47.6%)	

Data are described as mean ± SD or median (IQR) based on the parametric distribution

*S-C* Stone culture, *RP-C* Renal pelvis culture, *MSU-C* Midstream culture

Because of the presence of a significant association between S-C, RP-C, and MSU-C, three regression models were constructed using one of these factors combined with other significant predictors at once. On multivariate analysis, only positive S-C was the significant predictor of stone recurrence (odds ratio [OR] 9.9, 95% confidence interval [CI] [3.8–28.6], p < 0.001). For the RP-C model, only urine pH was the significant predictor for stone recurrence (OR 2.008, 95% CI [1.4–2.9], p < 0.001). Likewise, urinary pH was the only significant predictor in the MSU-C model (OR: 1.9, 95% CI [1.3–2.7], p = 0.001). Table 3 demonstrates the results of multivariate analysis.

**Table 3:** Multivariate logistic regression models for the predictors of stone recurrence

	OR	95%CI	P-value
S-C model			
(Intercept)	0.08	(0.001–4.9)	0.2
Positive S-C	9.9	(3.8–28.6)	< 0.001
GFR	1.04	(0.9–1.08)	0.07
Stone type: non-Ca containing	1.08	(0.4–3.2)	0.8
Urine citrate	1.001	(0.9–1.003)	0.5
Urine pH	0.8	(0. 5–1.4)	0.5
The RP-C model			
(Intercept)	0.001	(0–0.04)	< 0.001
Positive RP-C	1.3	(0.5–3.3)	0.6
GFR	1.03	(0.91–1.1)	0.1
Stone type: non-Ca containing	1.2	(0.4–3.3)	0.7
Urine citrate	1.001	(0.9–1.004)	0.1
Urine pH	2.008	(1.4–2.9)	< 0.001
MSU-C model			
(Intercept)	0.002	(0–0.06)	0.001
Positive MSU-C	1.7	(0.9–3.3)	0.1
GFR	1.03	(0.9–1.1)	0.2
Stone type: non-Ca containing	1.1	(0.4–3.1)	0.8
Urine citrate	1.001	(0.9–1.004)	0.2
Urine pH	1.9	(1.3–2.7)	0.001

*OR* Odds ratio, *CI* Confidence interval, *S-C* Stone culture, *RP-C* Renal pelvis culture, *MSU-C* Midstream culture

### Comparison of the three models

**Fig. 1:**
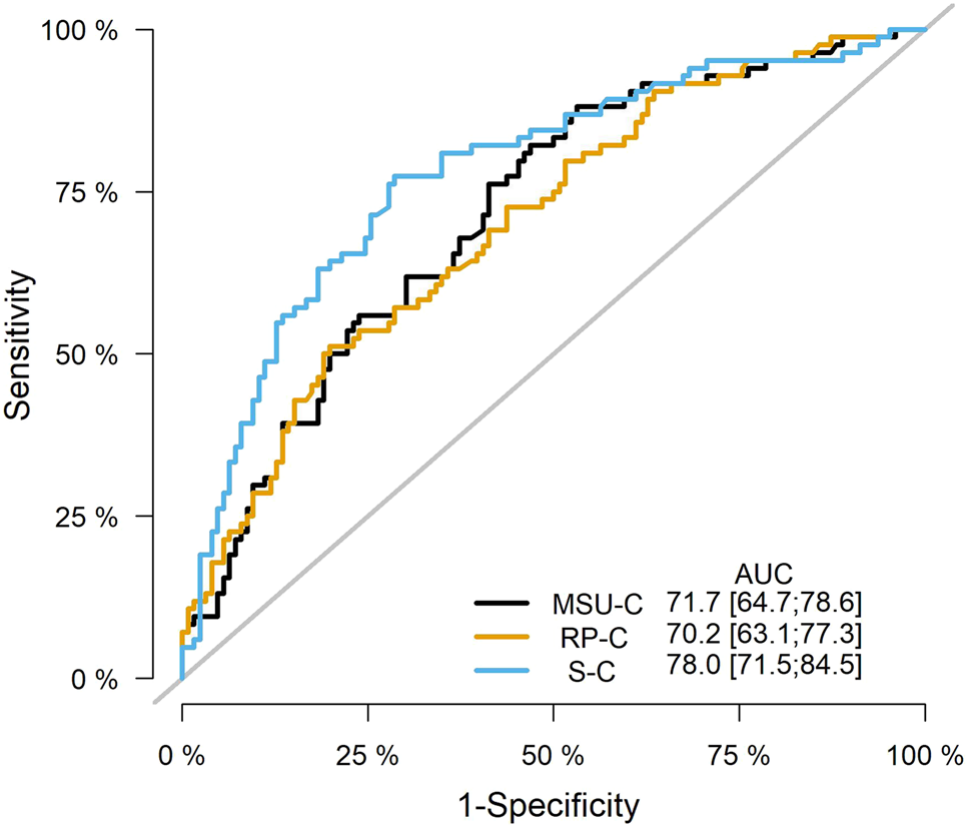
Receiver operating characteristic (ROC) curves for stone (S-C), renal pelvis (RP-C), and midstream urine (MSU-C) cultures multivariate logistic regression models with the area under the curve (AUC) and its 95% confidence intervals

Each model was evaluated using the ROC-derived AUC. The AUC of the S-C model was significantly higher than both the RP-C model (delta AUC 7.8 [2.6;13], p = 0.004), and MSU-C model (delta AUC 6.3 [0.9;11.8], p = 0.02). On the other hand, no significant difference was found between the AUC of RP-C and MSU-C models (delta AUC: – 1.5 [– 3.7;0.7], p = 0.2). Figure 1 shows the AUC with 95% CI of the three models.

### The levels of agreement

Cohen’s Kappa levels of agreement are demonstrated in Table 4. Fair to good levels of the agreement were found between RP-C and MSU-C, and between RP-C and S-C in the whole cohort. Likewise, it was also present between RP-C and MSU-C in the recurrence group and RP-C and S-C in the non-recurrence group.

**Table 4:** The levels of agreement between the midstream urine, renal pelvis, and the stone cultures

Patients	Renal pelvis culture	Stone culture	Overall comparison
All patients			0.43 (p < 0.001)
Midstream urine culture	0.45 (p < 0.001)	0.37 (p < 0.001)	
Renal pelvis culture		0.51 (p < 0.001)	
Recurrence group			0.37 (p < 0.001)
Midstream urine culture	0.51 (p < 0.001)	0.31 (p < 0.001)	
Renal pelvis culture		0.38 (p < 0.001)	
No recurrence group			0.44 (p < 0.001)
Midstream urine culture	0.35 (p < 0.001)	0.38 (p < 0.001)	
Renal pelvis culture		0.66 (p < 0.001)	

The level of agreement is interpreted based on Kappa level as > 0.75 (Excellent), 0.4–0.75 (Fair to good), and < 0.4 (Poor)

## Discussion

The hallmark findings of the current study are that positive S-C was the only independent significant factor associated with recurrent stone formation and that no metabolic stone workup parameter was significantly associated with stone recurrence. In addition, there was a discrepancy between the leading organism in S-C and RP-C (gram-positive *S. aureus*) compared to gram-negative *E. coli* in MSU-C.

The role of gram-positive and negative bacteria in promoting stone crystallization has been previously explored. Chutipongtanate and associates have shown that *E. coli*, *S. aureus*, *K. pneumoniae*, and *S. pneumoniae* dramatically promoted calcium oxalate crystal aggregation and growth to a diameter greater than the lumen of the distal tubules [15]. The authors noticed that this effect is specific to bacterial viability and is dose-dependent. In another report about PCNL for staghorn stones, recurrent episodes of UTIs were an independent predictor of stone recurrence or residual stone enlargement [14]. Likewise, the *Staphylococcus spp.* has been linked to staghorn stone recurrence [13].

In this report, S-C has achieved the most significant association with stone recurrence when compared to MSU-C or RP-C. To the best of our knowledge, the comparative effect of MSU-C, RP-C, and S-C has not been explored in the context of stone recurrence. The correlation between MSU-C, RP-C, and S-C has been extensively studied in evaluating postoperative sepsis with S-C being considered the most accurate tool [17]. MSU-C does not represent the infection status of the upper tract, especially in the presence of obstruction [18]. In addition, a weak correlation has been found between the lower urinary tract (MSU-C) and upper urinary tract (RP-C and S-C) [19]. Similarly, as a predictor of post-PCNL sepsis, S-C and RP-C have shown superior outcomes in the prediction of infectious complications in a recent meta-analysis [17].

The pivotal role of 24 h metabolic stone evaluation is to identify patients with urinary abnormalities that could specifically benefit from specific dietary recommendations and targeted medical therapy. This is why metabolic evaluation is recommended by the American Urological Association (AUA) as well as the European Association of Urology (EAU) [20]. On the other hand, contradictory results questioning this approach are emerging in concordance with our study. In a recent study using a propensity score matching analysis, 61.2% of patients who completed 24 h metabolic testing developed recurrent stone events compared to 54% who had not any metabolic evaluation (p < 0.001) [5]. Further analysis of patients with the recurrent stone disease showed that 57.1% of patients with metabolic evaluation developed a third stone-related episode compared to 53.3% of patients who had no metabolic evaluation (p < 0.001). More interestingly, testing the hypothesis of the significance of metabolic evaluation revealed that patients who had performed the metabolic evaluation and consequently received a new prescription of thiazide or alkali salt were more likely to develop another stone event compared to those who did not undergo metabolic evaluation or receive a prescription. In another report, Samson et al. examined the association between 24 h urine and stone recurrence in a large population of patients [10]. The authors reported that there was an annual decline in the usage of 24 h testing and that there was no significant association between performing the test and stone recurrence in either the total population or the high-risk groups.

Several factors could help interpret these results. Initially, problems related to the completion and accuracy of performing 24 h urine collection were cumbersome and exhausting, and some patients might not exhibit urinary abnormalities during the period of collection. In addition, there is no consensus on how clinicians interpret and treat the abnormalities if found. Furthermore, this metabolic evaluation should be followed by a strict diet regimen and a specific prescription which are less likely to be complied with by the patients [5]. 

Although our study shed the light on a new parameter that might affect stone recurrence and lessens the importance of another well-known factor, several drawbacks need to be acknowledged. Initially, the retrospective nature of the history of stone disease with the inability to document the primary stone burden, the inaccurate determination of the number of previous episodes, and the inability to investigate the history of UTI are potential factors that could have affected the outcome. Obese females constituted the main portion of our study compared to obese males (73.5 vs 53.8%, p = 0.006) which might not reflect the higher incidence of stone formation in males. The possible cause of this might be related to the geographical origin of this work where obesity is likely predominant and that the population demographics are changing with the growing incidence of obesity and metabolic syndrome. In addition, the examined metabolic evaluation and culture analyses were at the endpoint of the study. Therefore, the conclusion is better described as an association rather than a prediction. Furthermore, our study is missing the initial stone composition, stone density, and the initial metabolic evaluation and cultures which could have a significant impact on stone recurrence.

## Conclusion

Our results suggest that positive S-C outweighs the significance of metabolic stone abnormalities in patients with recurrent kidney stones. This highlights the significant role of UTI in the pathogenesis of stone recurrence. Our study suggests exerting all efforts to prevent further UTI that might prevent further stone recurrence. Prospective studies are highly encouraged to foresee the effect of preventing UTI on the chances of stone recurrence and to determine the actual role of metabolic workup.

## Abbreviations

BMI: Body mass index
MSU-C: Midstream urine culture
PCNL: Percutaneous nephrolithotomy
RP-C: Renal pelvis culture
S-C: Stones cultures
UTI: Urinary tract infection
